# Electrospun PVA/CuONPs/Bitter Gourd Nanofibers with Improved Cytocompatibility and Antibacterial Properties: Application as Antibacterial Wound Dressing

**DOI:** 10.3390/polym14071361

**Published:** 2022-03-27

**Authors:** Muhammad Nauman Sarwar, Hina Ghulam Ali, Sana Ullah, Kentaro Yamashita, Aiman Shahbaz, Umair Nisar, Motahira Hashmi, Ick-Soo Kim

**Affiliations:** 1 Nano Fusion Technology Research Group, Institute for Fiber Engineering (IFES), Interdisciplinary Cluster for Cutting Edge Research (ICCER), Shinshu University, Tokida 3-15-1, Ueda, Nagano 386-8567, Japan; nsoctober5@gmail.com (M.N.S.); sanamalik269@gmail.com (S.U.); 21fs326a@shinshu-u.ac.jp (K.Y.); motahirashah31@gmail.com (M.H.); 2 Faculty of Inorganic Chemistry, Karlsruhe Institute of Technology, Research Center Helmholtz Institute of Ulm (HIU), 89081 Ulm, Baden-Wurttemberg, Germany; hina.ali@kit.edu; 3 Department of Chemistry, Sargodha Campus, The University of Lahore, Sargodha 40100, Pakistan; aiman.shahbaz17@gmail.com; 4 Center for Solar Energy and Hydrogen Research, Faculty of Natural Sciences, Ulm University, 89075 Ulm, Baden-Wurttemberg, Germany; umair.nisar@uni-ulm.de

**Keywords:** nanofibers, antibacterial, copper nanoparticles, *Momordica charantia*, cytocompatibility

## Abstract

Antibacterial and cyto-compatible tricomponent composite electrospun nanofibers comprised of polyvinyl alcohol (PVA), copper II oxide nanoparticles (CuONPs), and *Momordica charantia* (bitter gourd, MC) extract were examined for their potential application as an effective wound dressing. Metallic nanoparticles have a wide range of applications in biomedical engineering because of their excellent antibacterial properties; however, metallic NPs have some toxic effects as well. The green synthesis of nanoparticles is undergoing development with the goal of avoiding toxicity. The aim of adding *Momordica charantia* extract was to reduce the toxic effects of copper oxide nanoparticles as well as to impart antioxidant properties to electrospun nanofibers. Weight ratios of PVA and MC extract were kept constant while the concentration of copper oxide was optimized to obtain good antibacterial properties with reduced toxicity. Samples were characterized for their morphological properties, chemical interactions, crystalline structures, elemental analyses, antibacterial activity, cell adhesion, and toxicity. All samples were found to have uniform morphology without any bead formation, while an increase in diameters was observed as the CuO concentration was increased in nanofibers. All samples exhibited antibacterial properties; however, the sample with CuO concentration of 0.6% exhibited better antibacterial activity. It was also observed that nanofibrous mats exhibited excellent cytocompatibility with fibroblast (NIH3T3) cells. The mechanical properties of nanofibers were slightly improved due to the addition of nanoparticles. By considering the excellent results of nanofibrous mats, they can therefore be recommended for wound dressing applications.

## 1. Introduction

Polyvinyl alcohol (PVA) is a nontoxic, biocompatible synthetic polymer. It is a colorless, odorless, water soluble polymer with good mechanical and thermal properties. The high versatility of its properties makes it suitable for many applications. For example, due to its biocompatibility and nontoxicity, it is a suitable polymer for biomedical applications. It has been already successfully used for the replacement of cartilage, contact lenses, wound dressing, etc. [1]. Extensive research has been conducted on PVA as a potential material for wound dressings. The use of PVA along with sodium alginate (SA) hydrogel, incorporated with PCL, has been reported recently as a wound dressing for burnt and cut wounds to enhance the healing process. Results of in vivo study in rats showed that fabricated wound dressings improved the regeneration of cell-induced tissues and enhanced the healing process of burnt wounds [2]. PVA, dextran, and chitosan crosslinked by glutaraldehyde has been reported by researchers as a potential wound dressing, especially for decubital ulcers. The results showed that a PVA, dextran, and chitosan-based wound dressing is antimicrobial, good cell proliferation and has a high water contact angle [3]. A PVA and sodium alginate (SA)-based wound dressing loaded with clindamycin drug has also been reported. The researchers claimed that the fabricated wound dressing healed the artificial wound on rats quickly, which was examined by in vivo testing, showing good drug release properties, mechanical properties, swelling ability, and thermal properties [4]. PVA/chitosan loaded with two different types of antiseptics (chlorhexidine (CHX) and polyhexanide (PHMB)) has been reported as a wound dressing. PVA/chitosan ratios were taken 1:1 and 3:1 wt.%. Results showed that the fabricated wound dressing with the 1:1 ratio showed good antibacterial activity [5].

Wound healing in the case of diabetic patients is always a challenge. To overcome this challenge, many researchers have offered their efforts. *Momordica charantia* (MC), commonly known as bitter gourd, is a well-known vegetable and abundantly found in Asia and Africa. It is famous for its medicinal characteristics and used for curing of different diseases such as diabetes, cancer, ulcers, HIV, and viral infections [6].

MC has potential application in the biomedical field. Its application has been reported in wound care and the synthesis of silver nanoparticles [7]. It also possesses anti-inflammatory properties. It has been reported as an excellent anti-inflammatory material through in vivo testing done on mice [8]. Natural materials and green vegetables have excellent biomedical applications due to their sustainability, biocompatibility, and green chemistry. MC has been reported as a potential candidate for bio-based synthesis of different metallic nanoparticles to enhance biocompatibility [9,10]. All parts of the MC plant were examined by researchers and reported as an excellent anti-inflammatory agent and can be used for biomedical applications such as wound dressing, diabetes and cancer treatment [7,11,12,13,14,15,16,17,18,19,20,21].

Electrospinning is easy, versatile, and is the finest spinning technique to fabricate smooth nanofibers with an excellent morphology. Both natural and synthetic polymers can be used to fabricate the nanofiber mats for several applications [22,23,24,25]. Electrospun nanofibers have found applications in different areas due to the wide range of easily electrospinable materials. Electrospun nanofibers can be used for environmental, electrical, electronics, food packaging, and biomedical applications [23,26,27,28,29]. Specifically, in the field of biomedical engineering, nanofibers have found applications in wound care, implants, skin care, tissue engineering, drug release, and biosensors [30,31,32,33,34].

Copper nanoparticles have excellent antimicrobial activity, antibacterial properties, and good mechanical and thermal stability which make it suitable for the healing of second degree burn wounds and breath masks [35]. Copper has two stable oxides. Copper (II) oxide is one of them. CuO nanoparticles also have applications in antiviral filters for influenza protection. In our recent research, CuO nanoparticles have been used for breath mask applications as an antimicrobial agent. CuO nanoparticles have exhibited excellent antibacterial properties; however, CuO nanoparticles were found to be slightly toxic when the loading percentage was increased, i.e., 1.00% (*w*/*w*). Therefore, current research is comprised of CuO nanoparticles supported by natural extract (*Momordica charantia*), which itself is a weak antibacterial agent; however, it is expected that the addition of MC extract will improve the cytocompatibility of CuO nanoparticles and will enhance their significance for biomedical and environmental applications. MC extract is also antibacterial, but it has a lack of mechanical strength, which is expected to be improved by CuO nanoparticles, as metallic nanoparticles tend to impart tensile strength to composites.

## 2. Materials and Methods

### 2.1. Materials

Polyvinyl alcohol (PVA) in the form of a polymer having a molecular weight in the range of 80,000–120,000, was purchased from Sigma Aldrich Corporation (St. Louis, MO, USA). The water soluble extract of *Momordica charantia* (MC) was purchased from Xi’an Qingzhi Biotechnology Co. (Shanghai, China). Nano-size powder of copper (II) oxide (<50 nm particle size), to be used as an antibacterial substance, was purchased from Sigma-Aldrich Corporation (Saint Louis, MO 63103, USA). Pure water and any additional necessary accessories were sourced from a chemical synthesis laboratory.

### 2.2. Methods

Electrospun nanofibers of PVA nanofibers were fabricated as detailed in our recent study [36]. PVA and MC were mixed at a ratio of 10% (*w*/*w*) in distilled water and stirred for 24 h (until a fully homogeneous solution was formed). Next, CuO was added in varying concentrations of 0, 0.2, 0.4, and 0.6 wt.% in spinning solution before electrospinning. The spinning solution was then electrospun to get nanofibers. The electrospinning solution was loaded into a plastic syringe, and the spinning conditions were as follows: the applied voltage was 15 kV, the tip to collector distance was 150 mm, the solution supply speed was 0.5 mL/h, the temperature was 20–25 °C, and the humidity was 55%. The operating parameters of electrospinning were kept the same for all samples. The electrospinning parameters were set according to our previous studies [25,37,38,39] and adjustments were made as required. Details of samples are given in Table 1.

### 2.3. Characterization

#### 2.3.1. Scanning Electron Microscopy (SEM)

The morphology analysis of all fabricated PVA/MC and PVA/MC/CuO nanofibers was conducted using a scanning electron microscope (SEM; JSM-6010LA, JOEL, Tokyo, Japan). Before studying the nanofibers’ morphology, the nanofibers were sputter-coated with platinum to make them conductive. A diameter of 50 random fibers from SEM by using Image-J software were calculated to analyze the average fiber diameter.

#### 2.3.2. SEM-EDS Analysis

The elemental mapping, focusing on Cu, was analyzed using an energy dispersive X-ray analyzer (EDS; JSM-6010LA, JOEL, Tokyo, Japan) integrated with a scanning electron microscopy (SEM).

#### 2.3.3. Transmission Electron Microscope (TEM)

The presence of CuO nanoparticles inside the produced nanofibers was confirmed using a transmission electron microscope (TEM), JEOL 2010 Fas TEM, Tokyo, Japan, which was accelerated with 200 kV. The samples were prepared by taking fibers from random places in the nanofibrous sheet. After that, a thin layer of fibers was placed onto the TEM-specific copper grids to take the TEM micrographs.

#### 2.3.4. Fourier Transform Infrared Spectroscopy-Attenuated Total Refraction (FTIR-ATR)

FTIR spectroscopy was used to investigate the molecular structure and any possible interaction between the PVA/MC and PVA/MC/CuO composites. Fourier transform infrared spectrophotometer (IR ATR Prestige-21, Shimadzu Co, Kyoto, Japan) with different wave measurement ranges from 500 cm^−1^ to 4000 cm^−1^ in total internal reflection measurement (ATR) mode and in the case of FTIR the range was kept from 400 cm^−1^ to 4000 cm^−1^.

#### 2.3.5. Moisture Vapor Transmittance Rate (MVTR) and Air Permeability (AP)

The air permeability of the prepared nanofibrous webs was measured using an air permeability tester (Kato Tech Co., Ltd., Osaka, Japan). Samples with a measurement of 2 × 2 cm were placed into the claws and air was run at a speed of 2 cm/s. The resistance of the webs against air was used to calculate air permeation of the samples. The moisture vapor transport rate (MVTR) for the same samples was measured using the upright cup method (A-2) for breathability measurement. The samples were placed in a chamber at 40 °C and a relative humidity of 50% for a period of 24 h. The weight difference was used to calculate the amount of water transported through the nanofibrous mats.

#### 2.3.6. Universal Testing Machine (UTM)

Mechanical strength is an important factor for examining the toughness and practical characteristics of nanofibers. The mechanical performances of the prepared samples of PVA/MC and PVA/MC/CuO were evaluated with a tensile test using a Tensilon universal material tester (RTC-1250A, A & D Co., Ltd., Tokyo, Japan). Four samples for each individual model were prepared following the ISO 13634 standard, and the experiment was performed at room temperature (25 °C ± 3 °C). The sample was pulled at a rate of 1 mm/min. The available UTM data was used to calculate stress and strain. The stress–strain curves were created and compared.

#### 2.3.7. Antioxidant Test

The antioxidant activity of the samples was measured by using a free radical of 2, 2-diphenyl minus 1-picryl hydroxyl (DPPH). A 100 mg sample was immersed in a 0.1 mM DPPH and methanol solution and incubated without light for 2 h under temperature control at 37 °C. In addition, a DPPH solution containing no sample was prepared as a control for comparison. The absorbance of the sample incubated at the above time was measured at 517 nm with a UV-Vis spectrophotometer (Jasco V-670). Antioxidant activity is expressed as a formula given below [40,41]. It was expressed as a percentage using the following equation:

(1) $$Antioxidant\;activity\;\left(\%\right)=\frac{A_{c}-A_{s}}{A_{c}}\times 100\;$$

where *A_c_* is the → control’s absorbance and *A_s_* is the sample’s absorbance.

#### 2.3.8. Antibacterial Test

Testing of the antibacterial properties of the prepared NF was carried out according to AATCC 147-1998, and the inhibition areas of *E. Coli* (*Escherichia coli*, BUU25113, gram negative) and *B. Subtilis* (*Bacillus subtilis* 168, gram positive) were investigated with the disk diffusion method and the antibacterial properties were evaluated. First, both *E. coli* and *B. Subtilis* were shake-cultured for 24 h. Both of the cultured bacteria samples were diluted with phosphate buffer 1.0 × 10^8^. A 100 µL sample of the bacterial solution, prepared as CFU/mL, was placed in a petri dish, 15 mL of agar medium was added, and the mixture was stirred and dried. Then, a sterilized sample disk (diameter: 10 mm) was prepared. The cells were placed into agar medium and cultured at 37 °C for 24 h. After that, by using the following Equation (2), with the diameter of the transparent circle (blocking circle) that blocked the growth of bacteria formed around the disc as C and the diameter of the sample disc as D, each sample became bacterial. The range of inhibition of reproduction (inhibition area I) was analyzed and the antibacterial properties were evaluated.

(2) $$I=\frac{C-D}{2}\;$$

#### 2.3.9. Cell Compatibility Test/Cytotoxicity Test (WST-1 Assay)

The in vitro cell proliferation was determined by utilizing a WST-1 colorimetric assay. NIH 3T3 mouse fibroblast cells were cultured in Dulbecco’s modified eagle medium (DMEM) supplemented with 10% fetal bovine serum (FBS) using a humidified incubator at 37 °C in a 5% CO_2_ atmosphere. The nanofiber mats were sterilized with a 70% ethanol solution and later washed with PBS. The sterilized mats were placed into a 96 well plate. The 96 wells culture plate was reduced to 60 wells by leaving the corner and edge wells blank to avoid the unnecessary evaporation of the media as the incubation time was prolonged (7 days). The well without mats were considered to be the control. The wells were seeded with 1000 cells/well in 200 µL of growth medium. The seeded wells were cultured for 1, 3, 5, and 7 days. At each of the time points (1, 3, 5, and 7 days), 10 µL of WST-1 was added to the corresponding well and further incubated for 2 h at 37 °C. The metabolically active NIH 3T3 cells were reacted with WST-1 reagent and produced formazan dye which was quantified at 450 nm using a microplate reader (Thermos scientific, Multiscan FC instrument). The cell viability was calculated as according to Equation (3). The measurement was performed 3 times.

(3) $$Cell\;viability\;\left(\%\right)=\frac{Abs_{sample}}{Abs_{control}}\times 100$$

Here, *Abs_control_* is a comparative well that does not include a sample.

## 3. Results

### 3.1. Morphological Properties

**Figure 1:**
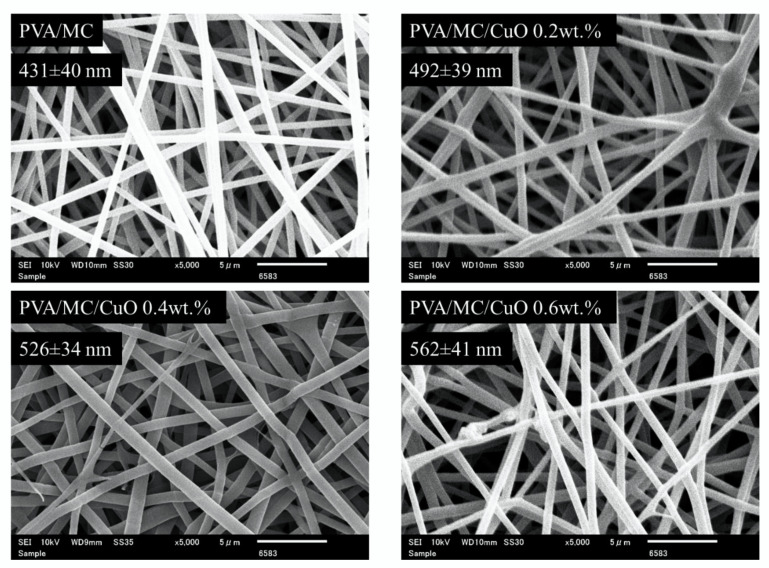
SEM images of PVA/MC, PVA/MC/CuO 0.2 wt.%, PVA/MC/CuO 0.4 wt.%, and PVA/MC/CuO 0.6 wt.% nanofibers.

SEM images of all fabricated nanofiber samples with increasing ratios of CuO until 0.6% (*w*/*w*) are shown in Figure 1. The SEM images indicate the morphological properties of the nanofibers. The nanofiber samples having PVA/MC are shown in Figure 1 which display smooth morphology with no bead observed in the image. The increase in the nanofiber’s diameter can be observed with the loading of CuO nanoparticles. As the concentration of CuO is increased, the average diameter of nanofibers were also increased, which can be easily observed in Figure 1. The prepared solution of PVA/MC was homogeneous, which reflects the smooth morphology of the nanofibers. The average fiber diameters of PVA/MC/CuO at CuO concentrations of 0, 0.2, 0.4, and 0.6 wt.% were 431 ± 40 nm, 492 ± 39 nm, 526 ± 34 nm, and 562 ± 41 nm, respectively. From this result, it was also confirmed that the average fiber diameter increased as the CuO concentration increased. It seems that the incorporation of CuO nanoparticles in all nanofibers spun in solution showed bead-free and uniform morphological properties. It was considered that the fiber system of these is enlarged by the entry of CuO nanoparticles into the nanofibers.

#### SEM-EDS Analysis

**Figure 2:**
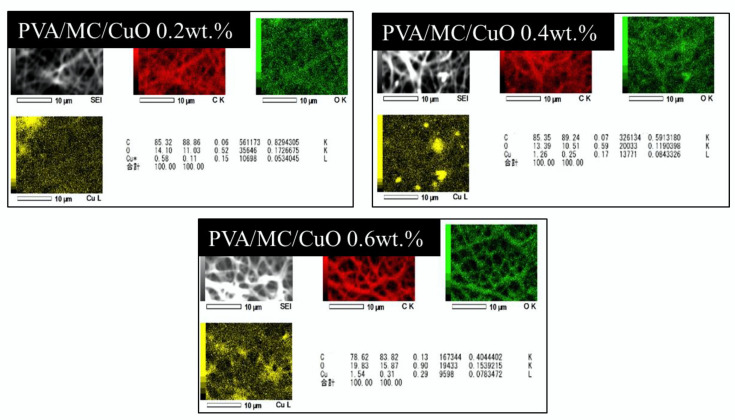
EDS analysis of PVA/MC/CuO 0.2 wt.%, PVA/MC/CuO 0.4 wt.%, and PVA/MC/CuO 0.6 wt.% nanofibers.

The results of SEM-EDS analysis are shown in Figure 2. Here, it was confirmed whether the CuO nanoparticles were incorporated into the produced nanofibers. It was determined that the samples having CuO nanoparticles shows a prominent value of Cu and O. In EDS analysis, it was observed that the electrospun nanofibers having 0.2%, 0.4%, and 0.6% CuO showed 0.58%. 1.26%, and 1.54% Cu, respectively. It was also observed that the amount of Cu in nanofibers was found to be linear with the initial concentration of CuO in the electrospinning solution, which represents the uniform mixing (suspension) of CuO nanoparticles in spinning solution.

### 3.2. FTIR (Fourier-Transform Infrared Spectroscopy)

**Figure 3:**
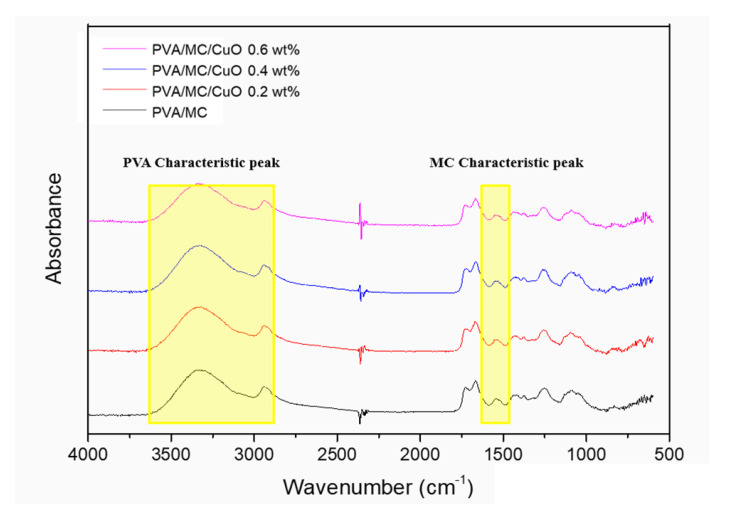
FTIR spectra of PVA/MC, PVA/MC/CuO 0.2 wt.%, PVA/MC/CuO 0.4 wt.%, and PVA/MC/CuO 0.6 wt.%.

Due to the abundance of hydroxyl groups, PVA is a hydrophilic polymer which makes PVA nanofibers unstable in water or aqueous medium. Before their practical use, PVA nanofibers were cross-linked by HCL and glutaraldehyde vapors, entangling the hydroxyl groups, making PVA nanofibers less hydrophilic [36,38,42]. Figure 3 shows the FT-IR spectra of the PVA/MC and PVA/MC/CuO nanofibers.

The peak around 2900 cm^−1^ is the vibration of -CH expansion and contraction, and the peak near 3300 cm^−1^ (3000~3500 cm^−1^) is the characteristic peak of the –OH hydroxyl group. Overall, these are the characteristic peaks of PVA confirmed in all nanofibers. The peaks around 1500 cm^−1^ to 1600 cm^−1^ are peaks characteristic of C=C expansion and the contraction of the benzene ring of the bitter gourd component. This peak was also confirmed in all nanofibers. From these results, it can be confirmed that both MC and PVA components can be synthesized in the produced nanofibers.

### 3.3. Transmission Electron Microscopy (TEM)

**Figure 4:**
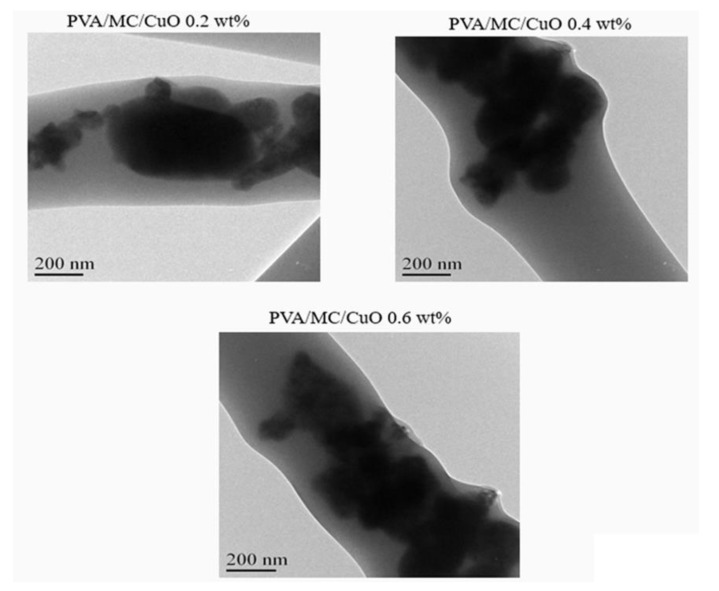
TEM images of the PVA/MC/CuO 0.2 wt.%, PVA/MC/CuO 0.4 wt.%, and PVA/MC/CuO 0.6 wt.% nanofibers.

Figure 4 displays the TEM image of PVA/MC/CuO nanofibers at 0.2 wt.%, 0.4 wt.%, and 0.6 wt.%. From Figure 4 it can be observed that the PVA/MC/CuO nanofibers presented the CuO nanoparticles dispersed in the nanofiber composite. It was confirmed by the resulting image that CuO nanoparticles were incorporated inside the nanofibers. Therefore, from the results of both the EDS analysis and the TEM image, the incorporation of CuO was confirmed.

### 3.4. Mechanical Properties (Tensile Test)

**Figure 5:**
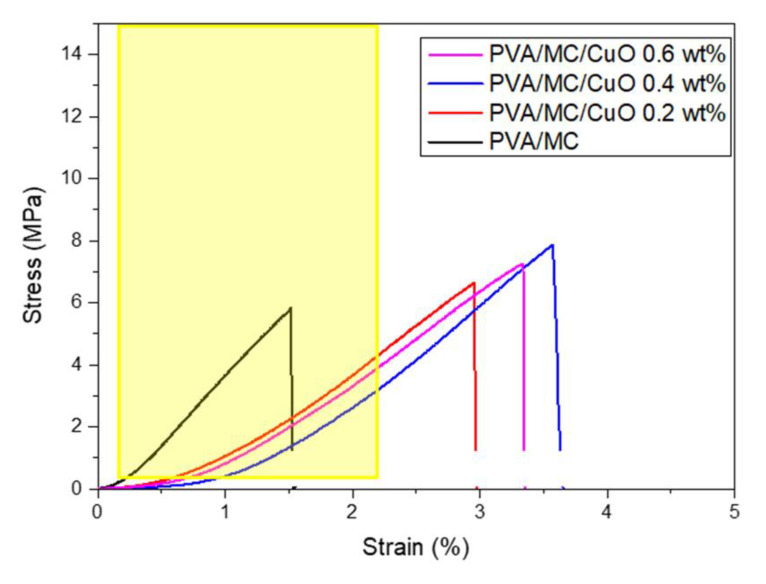
Stress–strain diagram of the PVA/MC/CuO 0.2 wt.%, PVA/MC/CuO 0.4 wt.%, and PVA/MC/CuO 0.6 wt.% nanofibers.

In the literature it is reported that the mechanical properties of the skin vary in strength in the range of 0.1 to 32 MPa and the strain ratio at fracture point varies in the range of 0.42 to 2.26 [43,44]. Therefore, it is necessary for the materials proposed for skin regeneration to retain properties that are reliable with those of the skin. However, there is non-uniformity in the structure of the skin, and the above values are not absolute values and depend on some properties such as genes, age, and skin color [43,44]. The mechanical properties of PVA/MC/CuO nanofibers were analyzed by a universal testing machine. The tensile strength, elongation at break, and tensile modulus were calculated from the stress strain data. Figure 5 shows the stress–strain curves of the PVA/MC nanofibers and PVA/MC/CuO nanofibers. It can be shown the maximum stress or tensile strength of pure the PVA/MC nanofiber mat is 5.82 MPa. A noticeable increase in the tensile strength of the PVA/MC/CuO nanofibers was observed and the maximum stress of the nanofibers carrying CuO was 7.26, 7.93 and 7.69 MPa at 0.2 wt.%, 0.4 wt.% and 0.6 wt.%, respectively. This indicates that addition of CuO nanoparticles adds good mechanical properties to the nanofiber. Based on a previous study, which showed that a material having a mechanical property which allows a curve to pass through the above range (i.e., 4 MPa) is a property that can be sufficiently used as a wound dressing, the nanofibers carrying CuO produced here can be applied as a wound dressing.

### 3.5. Antioxidant Test

**Figure 6:**
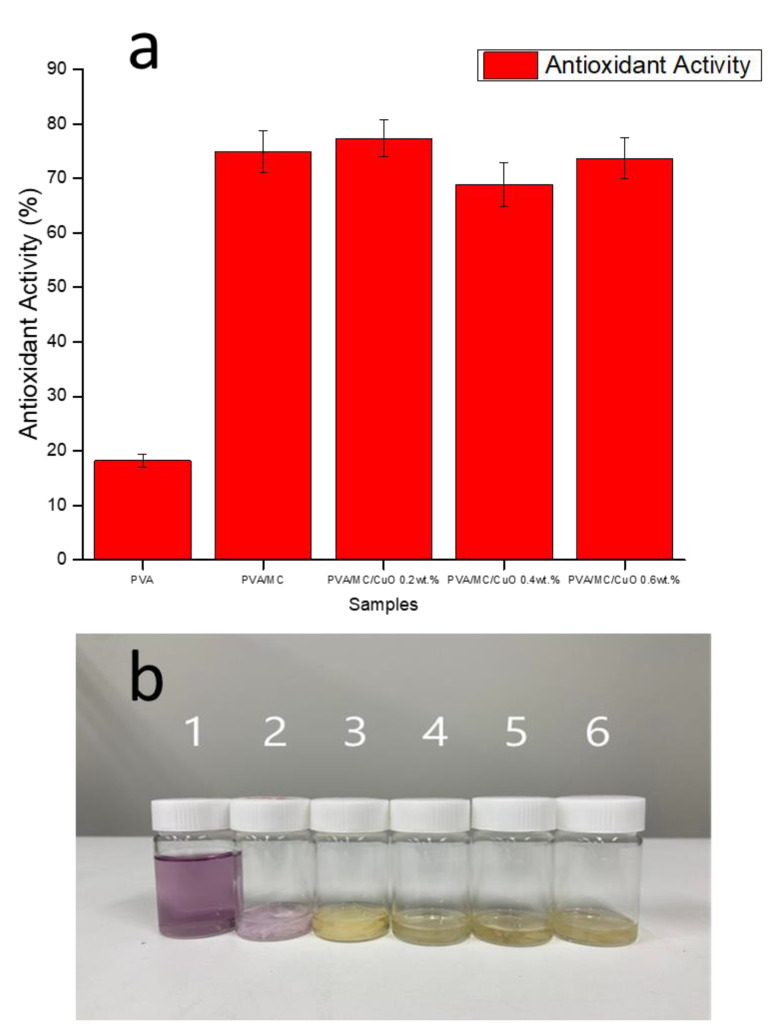
(**a**) DPPH free radical scavenging activity of PVA/MC/CuO 0.2 wt.%, PVA/MC/CuO 0.4 wt.%, and PVA/MC/CuO 0.6 wt.%. (**b**) Color appearances of 1—DPPH free radical scavenging activity, 2—PVA, 3—PVA/MC, 4—PVA/MC/CuO 0.2 wt.%, 5— PVA/MC/CuO 0.4 wt.%, 6— PVA/MC/CuO 0.6 wt.%.

Figure 6a shows the results of the DPPH free radical test which was performed to calculate the antioxidant activity of each produced nanofiber. This test confirmed the antioxidant activity of the phenol present in the bitter gourd components. As shown in Figure 6b, when a substance with strong antioxidant activity is placed in a DPPH solution, its color changed to yellow. This discoloration was measured using a UV-Vis spectrophotometer, and the free radical inhibition rate was also measured. The pure PVA nanofiber solution without MC did not change to a yellow color. Since the solution of nanofibers containing other MCs changed to yellow, it confirmed the antioxidant activity of the phenol group enclosed in bitter gourds. Since the hydroxyl group is present in the structure of the pure PVA nanofiber, it showed slight antioxidant activity, even if it did not contain MC.

### 3.6. Moisture Vapor Transmittance Rate (MVTR) and Air Permeability (AP)

**Figure 7:**
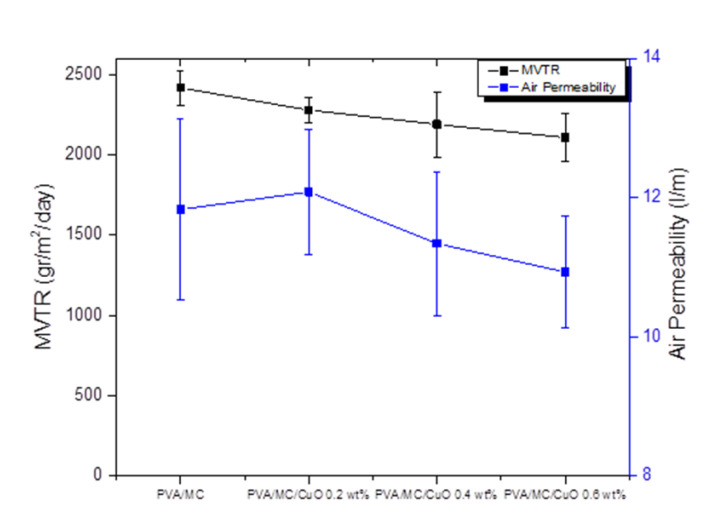
Air permeability and moisture vapor transport rate of the prepared nanofibers.

Air permeability is an important parameter, along with moisture vapor transport rate (MVTR), to assess the performance of wound dressing. Figure 7 shows that both AP and MVTR were slightly decreased with the addition of CuO nanoparticles (CuONPs). Air permeability for PVA/MC nanofibers were 11.83 ± 1.3 L/m, while the air permeabilities for PVA/MC/CuO 0.2%, PVA/MC/CuO 0.4%, and PVA/MC/CuO 0.6% were measured as 12.08 ± 0.9, 11.34 ± 1.03, and 10.93 ± 0.8 L/m, respectively. Considering the impact of the standard deviation, it can be said that the air permeability of all samples was similar. Additionally, it was confirmed in the literature that air permeability over 6 L/m was considered to be in a good range, and the nanofibrous samples showed better air permeation when compared to films. In the same figure, a plot of MVTR is also shown, which also shows a similar trend as that of air permeability. It was observed that all samples exhibited MVTR in the range of 2100–2400 gr/m^2^/day, with a decreasing trend as the amount of CuONPs increased. The decrease in air permeability and MVTR may be due to the addition of CuO, as the presence of CuO may block the pores among nanofibers. When the concentration of CuO was kept to a lower level (to prevent toxic effects), the change in AP and MVTR was also negligible.

### 3.7. Antibacterial Test

The antibacterial properties of the prepared PVA/MC and PVA/MC/CuO nanofibers were observed by testing the antibacterial activity against Gram-negative *Escherichia coli* and Gram-positive *Bacillus subtilis* using the disk diffusion method [38,39,45,46]. We chose Escherichia coli and *Bacillus subtilis* in this study on the basis that both *Escherichia coli* and *Bacillus subtilis* are among the major bacteria that exist in the living environment. Pictures were taken and Image J software was used to calculate the zone of inhibition. 

**Figure 8:**
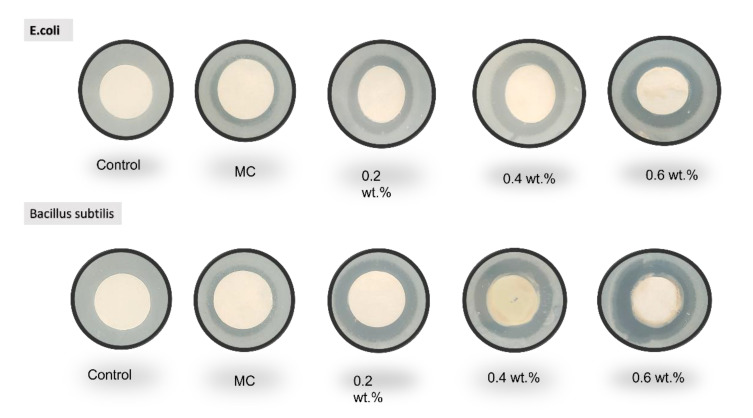
Disk diffusion test of PVA/MC (CuO 0.0%), PVA/MC/CuO 0.2 wt.%, PVA/MC/CuO 0.4 wt.%, and PVA/MC/CuO 0.6 wt.%. nanofibers.

Figure 8 displays that neat PVA/MC nanofiber are weakly antibacterial and showed weak antibacterial activity against bacterial stain types (Gram positive and Gram negative), while PVA/MC/CuO nanofibers with different concentrations of CuONPs showed excellent antibacterial activity. In addition, it was concluded from the range of inhibition that the samples have better antibacterial activity against Gram-positive *Bacillus subtilis* bacteria as compared to that of Gram-negative *Escherichia coli*. The main reason for this difference is the different cell wall structure of both types of bacteria. A similar trend of antibacterial activity for both types of bacteria was also observed in previous studies [23,25,38,47]. The inhibition zone for PVA/MC, PVA/MC/0.2% CuO, PVA/MC/0.4% CuO, and PVA/MC/0.6% CuO samples were recorded as 2.1 mm, 2.9 mm, 3.6 mm, and 3.12 mm for Gram-positive (*B. Sub*) bacteria, respectively, and 1.37 mm, 2.24 mm, 2.74 mm, and 2.98 mm for Gram-negative *(E. coli*) respectively.

### 3.8. Cytocompatibility Test

**Figure 9:**
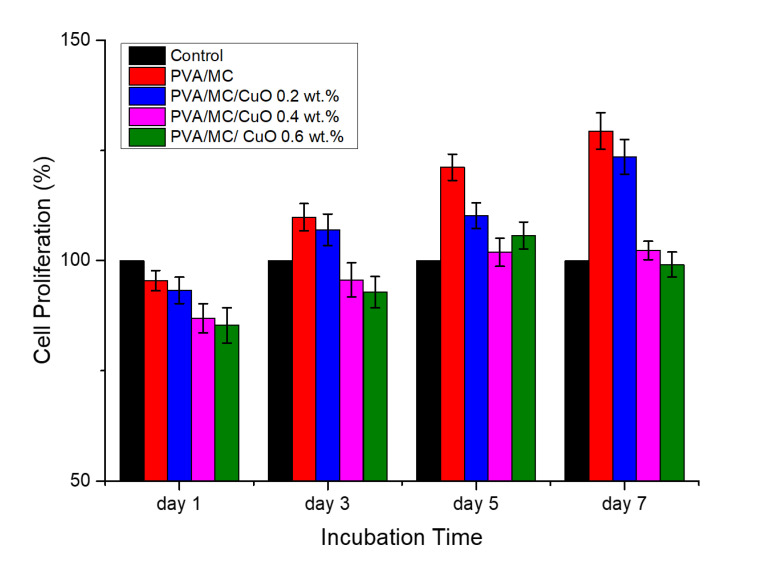
The cell viability of samples after one, three, five and seven days.

A WST-1 assay was used to assess cell proliferation and the results of the cell viability measurement are shown in Figure 9. Using WST-1 colorimetry, mouse fibroblasts and nanofibers were in contact for 7 days to analyze cell compatibility. For comparison, the cell viability of the wells tested without using samples was 100% (control). According to ISO 10993-5, a cell viability value above 80% is nontoxic, 60–80% is weakly toxic, 40–60% is addictive, and less than 40% is highly toxic. From Figure 9, all of the samples displayed a cell viability exceeding 80% from day 1 to day 7, and all samples exceeded 100% on day 5. From these results, it was confirmed that all the produced nanofibers have excellent cell compatibility. It could be possible that all of these nanofiber non-woven fabrics are non-toxic to the human body and can be applied in wound dressing applications. The main purpose of this study was to improve the cytocompatibility of electrospun nanofibers containing CuO nanoparticles. As in our previous study regarding utilization of CuO as an antibacterial agent, it was observed that nanofibers containing CuO were slightly toxic, as cell proliferation was decreased to 50% [25]. In a study using only *Momordica charantia* as an antibacterial agent, it was observed that nanofibers were good in cytocompatibility while weakly antibacterial [38]. Considering the pros and cons of both types of materials, this study was proposed to optimize the antibacterial and cytocompatibility of nanofibers using composite of CuO nanoparticles and *Momordica charantia* extract. It can be observed by the results of the cytocompatibility test that all these nanofibrous mats were found to be non-toxic.

## 4. Conclusions

PVA/MC nanofibers were successfully electrospun with varying concentrations of CuO in nanofibers. The ensile strength of PVA/MC nanofibers was 5.82 MPa, and the maximum stress of nanofibers carrying 0.4 wt.% CuO was considerably improved to 7.93 MPa. The addition of CuO nanoparticles gave strength to the PVA/MC nanofibers. However, it was a slight increase and when considering the standard deviation, it can be concluded that the addition of nanoparticles caused a very small strength gain. PVA/MC/CuO nanofibers also exhibited uniformity in morphological properties (bead-free nanofibers). The synthesized nanofibers incorporating CuO nanoparticles at a concentration of 0.6 wt.% were superior to other samples in wound dressing. Prepared nanofibers also offered outstanding properties in regard to cell compatibility and antioxidant/antibacterial activity. Comparing the results of previous studies using CuO nanoparticles and *Momordica charantia*, it can be observed that this study provides better cyto-compatible and antibacterial scaffolds, having cell proliferation above 100% when compared to that of the control. Antibacterial activity was also improved against both types of bacteria (*E. Coli* and *B. Subtilis*). Taking into account all of the results, it can be concluded that prepared scaffolds may be used as an effective antibacterial wound dressing.

## Figures and Tables

**Table 1:** Sample codes with concentrations of PVA, MC, and CuONPs.

Samples	PVA	MC	CuONPs
PVA/MC	10	10	0
PVA/MC/CuO 0.2 wt.%	10	10	0.2
PVA/MC/CuO 0.4 wt.%	10	10	0.4
PVA/MC/CuO 0.6 wt.%	10	10	0.6

## Data Availability

The data presented in this study are available on request from the corresponding author.
